# A New Method for Multipath Filtering in GPS Static High-Precision Positioning

**DOI:** 10.3390/s19122704

**Published:** 2019-06-16

**Authors:** Ke Han, Canyang Tang, Zhongliang Deng

**Affiliations:** School of Electronic Engineering, Beijing University of Posts and Telecommunications, No. 10 Xitucheng Road, Beijing 100876, China; hanke@bupt.edu.cn (K.H.); dengzhl@bupt.edu.cn (Z.D.)

**Keywords:** two-dimensional moving weighted average algorithm, multipath, wavelet packet algorithm, GPS static high-precision positioning

## Abstract

It is well known that multipath is one of the main sources of errors in GPS static high precision positioning of short baselines. Most algorithms for reducing multipath manipulate the GPS double difference (DD) observation residuals as input signal in GPS signal processing. In the traditional multipath mitigation methods, applying the wavelet transform (WT) to decompose the GPS DD observation residuals for identifying the multipath disturbance cannot effectively filter out the white noise of the high frequency part of the signal, and it is prone to edge effect. In this paper, for extracting multipath, a wavelet packet algorithm based on two-dimensional moving weighted average processing (WP-TD) is proposed. This algorithm can not only effectively filter out the white noise of the high frequency part of the signal, but also weaken the influence of the edge effect. Furthermore, considering the repeatability of multipath error in static positioning, we propose a method for determining the level of wavelet packet decomposition layers which make multipath extraction more effectively. The experimental results show that the corrected positioning accuracy is 14.14% higher than that of the traditional wavelet transform when applying the obtained multipath to DD coordinate sequences for position correction.

## 1. Introduction

In GPS static high-precision positioning of short baselines, errors such as ionospheric delay, tropospheric delay, satellite orbit error, receiver clock error, and satellite clock error can be eliminated or attenuated by differential techniques while multipath disturbance are not correlated at both ends of the baseline which make it impossible to be eliminated by differential techniques and become the main source of error affecting the positioning accuracy [1,2].

GPS multipath disturbance occurs when GPS signals travel from a satellite to a receiver via several paths due to reflection or diffraction of signals by nearby obstacles [3]. Multipath can distort signal modulation and carrier phase, thereby reducing the accuracy of GPS static high-precision positioning. In addition, this effect may hinder the fixation of ambiguities and even lead to erroneous solutions.

In order to mitigate and eliminate multipath, a variety of different strategies have been proposed, including appropriate site selection, hardware-based methods and software-based methods.

Appropriate site selection is the simplest and the most effective way to solve the multipath effect. The construction of the base station should be chosen in an open area, avoiding various obstacles causing excessive reflection and obtaining as many visible satellite signals as possible.

PozoRuz et al. (1998) proposed a method calculating the positions in the plane using the three highest satellites based on a new satellite selection criterion which can reduce multipath [4].

The hardware-based method refers to improvements of antenna and receiver design.

Scire Scappuzzo et al. (2009) designed a low-multipath wideband GPS antenna with cutoff or non-cutoff corrugated ground plane which can operate uniformly between 1.15 GHz and 1.60 GHz while maintaining the required low-multipath performance in the whole bandwidth [5]. Ray et al. (1999) developed a system for reducing the effect of carrier-phase multipath on static GPS applications using multiple closely spaced antennas [6]. Bryan R. Townsend et al. (1994) introduced a new tracking loop which takes full advantage of the Narrow Correlator spacing design, but, in addition, is much more resistant to multipath effects on the correlation function and thereby reduces the multipath bias on the pseudorange measurements [7]. R.D.J. van Nee et al. (1992) designed a specific receiver structure which simultaneously estimates the parameters of line-of-sight plus multipath signals for reducing GPS code and carrier multipath errors [8].

However, the hardware is inaccessible for most users and it is expensive. Despite this, we cannot deny that hardware methods have better performance in other aspects, such as stability and efficiency. In contrast, software methods are more convenient and cheaper for most people.

The software-based method mainly focuses on the post-processing of signal by using algorithms and filters.

Axelrad et al. (1996) proposed an improved technique which can adaptively estimate the spectral parameters (frequency, amplitude, phase offset) of multipath in the associated signal-to-noise ratio (SNR) [9]. Linlin Ge et al. (2000) proposed an adaptive finite-duration impulse response filter, based on a least-mean-square algorithm, that has been developed to derive a relatively noise-free time series from the CGPS results [10]. There are some other filters, such as Kalman filters (Nce and Sahin 2000) [11], FIR filters (Han and Rizos (1997)) [12], and Vondrak filters (Vondrak (1977) [13], have also been developed to reduce GPS multipath effects.

In addition, wavelet transform has a good localization property within both the time and frequency domains. Particularly, after Stephane Mallat proposed the multi-resolution algorithm, also named Mallat algorithm [14], wavelet transform is widely applied signal processing, including extracting multipath.

Huang et al. (2003) applied wavelet transform in dynamic deformation monitoring for high-rise buildings [15]. Zhang and Bartone (2004) applied the wavelet technique to mitigate errors for satellite based navigation systems which can mitigate multipath error in a real-time conditions [16]. E. M. Souza, J. F. G. Monico (2004) apply the wavelet transform to decompose the pseudorange and carrier phase DD signals to separate the high frequencies, which are due to multipath from long delays, and the low frequencies effects, associated with multipath from short delays [17]. P. Zhong et al. (2008) proposed a method based on the technique of cross-validation for automatically identifying wavelet signal layers is developed and used for separating noise from signals in data series, and applied to mitigate GPS multipath effects [3]. M. R. Azarbad and M. R. Mosavi (2014) proposed a new multipath mitigation method based on the wavelet transform (WT). The method uses the stationary wavelet transform (SWT) to decompose the double difference residuals [18]. Lawrence Lau (2017) proposed a generic and robust three-level wavelet packets based denoising method for repeat-time-based carrier phase multipath filtering in relative positioning; the results show that the wavelet packets based method is better than the DWT-based method in the repeat time-based multipath filtering [19]. Souza et al. (2017) proposed a new approach for structure monitoring from GPS multipath effect and wavelet spectrum, and the experience investigated the feasibility of using wavelet spectra analysis of the multipath signal to monitor structure movement [20].

As we know, based on the Mallat algorithm, a signal can be decomposed into scale space and wavelet space. With the increase of the decomposition levels, the scale space would be decomposed continuously, whereas the wavelet space cannot be decomposed, which results in a low resolution in high frequency regions of wavelet transform [21]. This means that the multipath in the high frequency part of the signal cannot be effectively extracted by traditional wavelet transform.

Hence, wavelet packet transform (WPT) was pioneered by Coifman et al. based on wavelet transform [22]. WPT keeps the property of wavelet transform naturally, good localization property within both the time and frequency domains. This property greatly improves the accuracy of WPT in analyzing non-stationary and non-periodic signals. In addition, WPT effectively mitigates the defects in wavelet transform of low resolution in high frequency regions by decomposing both the scale space and wavelet space [21]. This means we can further subdivide the high frequency portion of signal and extract multipath more effectively.

The multipath extraction efficiency partially depends on the chosen mother wavelet for WPT. In this paper, we won’t attempt to compare the performance of mother wavelets on retrieving multipath errors from noisy coordinate residual sequences. E. M. Souza et al. (2004) had verified that SYM12, SYM8 and DAUB8 presented the better performance for reconstruction of the GPS DD signal [17]. In this paper, we will choose DAUB8 as the mother wavelet for WPT.

The other challenging issue related to the multipath extraction efficiency is the level of decomposition which depends on the sub-band frequency in which multipath lies [18]. Thence, we also propose a method for determining the number of wavelet packet decomposition levels considering the repeatability of multipath error in static positioning.

In addition, edge effects in signal processing using wavelet packet transform is another un-neglected problem. P. Zhong et al. (2008) choose about 70% of the data in the middle of the observational series for cross-validation to prevent edge effects due to poorer filtering results at the ends of a data series [3]. This method solves the edge effect problem in a certain condition, but, when the coordinate sequence residual is short, it will become invalid. Therefore, referring to the idea of moving weighted average method and the principle of bilateral filters in image processing [23,24], we propose a new algorithm named the two-dimensional moving weighted average algorithm. The double-difference coordinate residual sequences are smoothed by the proposed algorithm to achieve the purpose of edge-preserving and pre-denoising.

In general, we propose a new method named wavelet packet algorithm based on two-dimensional moving weighted average processing (TDMWA), compared with the traditional wavelet algorithm, which can not only more effectively mitigate the multipath of the DD coordinate residual sequences, but also effectively weaken the influence of the edge effect.

This study is organized as follows. The principle of position correction based on multipath periodicity is introduced in the Section 2. The theory of the two-dimensional moving weighted average algorithm and the preprocessing of smoothing the double-difference coordinate sequence residual obtained by static observation for three consecutive days using TDWMA algorithm are introduced in Section 3. The theory of wavelet packet transform, the theory of correlation analysis of smoothed double-difference coordinate residual sequences, the specific steps for determining the number of wavelet packet decomposition layers and the principle of wavelet packet threshold denoising will be introduced in Section 4. The overall program of mitigating the multipath in GPS static high-precision positioning will be introduced in Section 5. The experimental verification will be introduced in Section 6 and Section 7. The data set used in Section 6 is the simulation data for verifying the feasibility of the proposed method. Section 7 uses the GPS data acquisition system built to collect the GPS data to further verify the feasibility of the actual environment and the denoising performance of the Vondrak algorithm, traditional WT algorithm and the WP-TD algorithm is compared from the correlation and root mean square error. Conclusions are given in Section 8.

## 2. The Principle of Position Correction Based on Multipath Periodicity

In this paper, we take the double difference carrier phase observation residuals obtained from the reference day (Day 1) as the correction to the observations on the adjacent days (Day 2 and Day 3) considering that multipath is repetitive in static observations of the fixed point. Methods for position correction based on multipath repeatability can be found in Khelifa et al. (2011) [25], Ye et al. (2013) [26], and Lawrence Lau et al. (2017) [19].

The double difference carrier phase observations [27] indicated as Equation (1):

(1) $$\Phi_{rb}^{nm}=\lambda^{-1}\rho_{rb}^{nm}+N_{rb}^{nm}+\varepsilon_{\phi ,rb}^{nm}+M_{\phi ,rb}^{nm},$$

where $\Phi_{rb}^{nm}$ denotes the double difference carrier phase observation between satellites *n* and *m*, and stations *r* and *b*. $\rho_{rb}^{nm}$ denotes the geometric distance from the satellite center to the antenna phase center. $N_{rb}^{nm}$ denotes the double difference integer ambiguity. $\varepsilon_{\phi ,rb}^{nm}$ denotes the double difference carrier phase measurement noise. $M_{\phi ,rb}^{nm}$ denotes the double difference carrier phase multipath. λ denotes the carrier wavelength.

In GPS high-precision relative positioning of short baseline (less than 3 km). Since the DD carrier phase multipath error is always less than a quarter of the carrier wavelength, the observation residuals obtained on the reference day do not take the double difference integer ambiguity into consideration [21].

Based on the double difference carrier phase observations, the positioning solution of each epoch on the reference day indicated as Equation (2):

(2) $${\left[\dot{X}\right]}_{Re}=\left[\bar{X}\right]+{\left[M\right]}_{Re}+{\left[\varepsilon \right]}_{Re},$$

where $\dot{X}$ denotes the best estimated positioning solution of the fixed point in static observation. $\bar{X}$ denotes the known positioning solution of the fixed point in static observation. M denotes the multipath, and ε denotes the observation noise (white noise). Subscript Re indicates the reference day.

Moving the known item of Equation (2) to the left side to get Equation (3):

(3) $${\left[\dot{X}\right]}_{Re}-\left[\bar{X}\right]={\left[M\right]}_{Re}+{\left[\varepsilon \right]}_{Re}.$$

The right side of the Equation (3) is the positioning residuals that consist of multipath and observation noise (white noise), which will be regarded as the DD coordinate residual sequences in the next section.

Because the repeat-time-based multipath increases the observation noise level in the positioning solutions of the fixed point on the adjacent days, it is necessary to eliminate the noise of the DD coordinate residual sequences and obtain the noiseless multipath as the correction of the positioning solutions of the adjacent days.

The corrected position of each epoch on the adjacent day indicated as Equation (4):

(4) $${\left[\hat{X}\right]}_{Ad}+{\left[\varepsilon \right]}_{Ad}={\left[\dot{X}\right]}_{Ad}-{\left[M\right]}_{Re},$$

where $\hat{X}$ denotes the multipath-corrected positioning solutions. Subscript Ad indicates the adjacent days.

## 3. Preprocessing of the DD Coordinate Residual Sequences by the TDMWA Algorithm

### 3.1. The Basic Principle of the TDMWA Algorithm

In GPS static high-precision positioning, the multipath tends to be stable between the adjacent epochs, but the DD coordinate residual sequences fluctuation intensified between adjacent epochs due to the existence of white noise. Even worse, there exist poorer filtering results at the end of the DD coordinate residual sequences because of the edge effects.

In order to weaken the influence of edge effects and weaken the white noise, we propose a new method named two-dimensional moving weighting average algorithm (TDMWA) for smoothing the DD coordinate residual sequences. Considering that the white noise is independent and obeys the normal distribution, the algorithm performs moving weighted average processing on each of the observation epoch in the time domain and the value domain, respectively, which can be described as the following formulation equation:

(5) $$\{\begin{array}{c} X_{r}^{T}=\frac{\alpha_{\left[\frac{T}{2}\right]}x_{j-\left[\frac{T}{2}\right]}+\alpha_{\left[\frac{T}{2}\right]-1}x_{j-\left[\frac{T}{2}\right]+1}+\ldots \ldots \alpha_{0}x_{j}+\alpha_{1}x_{j+1}+\ldots \ldots +\alpha_{\left[\frac{T}{2}\right]}x_{j+\left[\frac{T}{2}\right]}}{T}\\ X_{r}^{N_{r}}=\frac{\sum_{k=1,x_{k}\in \mathbb{Q}}^{N_{r}}\beta_{k}x_{k}}{N_{r}} \end{array},$$

where $X_{r}^{T}$ is the time domain component of the original DD coordinate residual sequence of the *r*-th observation epoch, and T is the moving average period of the time domain component, taken as an odd number, and T≤I (I is the total number of observation epochs). $X_{r}^{N_{r}}$ is the value domain component of the original DD coordinate residual sequence of the *r*-th observation epoch, and $N_{r}$ is the moving average period of the value domain component, and $N_{r}\leq I$, *r* represents the current epoch, and r∈I.

[] denotes an integer, $x_{j}$ is the decentralized epoch coordinate of the time domain component which determines the smoothed DD coordinate residual of each observation epoch in the time domain, $x_{k}$ is the decentralized epoch coordinate of the value domain component which determines the smoothed DD coordinate residual of each observation epoch in the value domain, and ℚ is the set consisting of the decentralized epoch coordinates.

$\alpha_{j}$ is the weighting coefficient of the *j*-th delay epoch in the moving average period T, and $\beta_{k}$ is the weighting coefficient of the *k*-th decentralized epoch in the moving average period $N_{r}$, then

(6) $$\{\begin{array}{c} \alpha_{j}=\alpha_{0},\alpha_{1},\ldots ,\alpha_{\left[\frac{T}{2}\right]},\alpha_{0}+2(\alpha_{1}+\ldots \alpha_{\left[\frac{T}{2}\right]})=1\\ \beta_{k}=\beta_{1},\ldots ,\;\beta_{\left[N_{r}\right]},\beta_{1}+\ldots +\beta_{\left[N_{r}\right]}=1 \end{array}.$$

### 3.2. The Process of Smoothing the DD Coordinate Sequence Residual by the TDMWA Algorithm

#### 3.2.1. Determine the Moving Average Periods T and the Decentralized Epoch Coordinate $x_{j}$ of Time Domain Component

Wherein the time domain components have the same moving average period T, and T is consistent with the influence period of the multipath effect.

The current epoch coordinate $x_{r}$ is taken as the median value of the corresponding decentralized epoch coordinate of the *r*-th observation epoch and the number of the corresponding decentralized epochs is *T*.

#### 3.2.2. Determine the Moving Average Periods $N_{r}$ and the Decentralized Epoch Coordinate $x_{k}$ of Value Domain Component

Based on the idea of bilateral filters in image processing, the moving average period $N_{r}$ of each value domain component and the corresponding decentralized epoch coordinate $x_{k}$ is determined according to the discrete distribution of the observation epochs.

Firstly, we need calculate the average value of all the observation epoch coordinate ${\bar{x}}_{I}$

(7) $${\bar{x}}_{I}=\frac{\sum_{i=1}^{I}x_{i}}{I},$$

where $x_{i}$ represents the all observation epoch coordinates, and i∈I.

Then, calculating the standard deviation of the observation epoch coordinate, and using the standard deviation as the threshold $\theta_{I}$ for selecting decentralized epoch coordinate constituting ℚ:

(8) $$\{\begin{array}{c} \sigma_{I}=\frac{\sum_{i=1}^{I}{\left(x_{i}-{\bar{x}}_{I}\right)}^{2}}{I}\\ \theta_{I}=\sigma_{I} \end{array}.$$

Then, the value of the current observation epoch coordinate $x_{r}$ becomes the new mean of all the observation epochs,

(9) $${\bar{x}}_{I}=x_{r},r\in I.$$

Next, calculating the standard deviation $\sigma_{i}$ of all the observation epochs coordinate $x_{i}$ according to the new mean obtained in Equation (9),

(10) $$\sigma_{i}=\frac{\sum_{i=1}^{I}{\left(x_{i}-x_{r}\right)}^{2}}{I}.$$

The observation epoch coordinate whose standard deviation $\sigma_{i}$ is not greater than the threshold $\theta_{I}$ is used as the decentralized epoch coordinate $x_{k}$, which will be selected to determine the smoothed value of the *r*-th observation epoch in the value domain, and the number of the decentralized epoch coordinate $x_{k}$ is the moving average period $N_{r}$, and $N_{r}\leq I$.

#### 3.2.3. Determine the Weighting Coefficient $\alpha_{j}$ and $\beta_{k}$

In the time domain, calculating the epoch delay of each decentralized epoch coordinate $x_{j}$ and the current observation epoch coordinate $x_{r}$, and weighting the corresponding decentralized epoch coordinate $x_{j}$ according to the size of the delayed epoch, the smaller the epoch delay, the greater the influence of the proximity effect, and the greater the weight of the corresponding decentralized epoch coordinate $x_{j}$.

Let $\bar{\tau }$ be the average epoch delay of each decentralized epoch coordinate $x_{j}$ relative to the current observation epoch $x_{r}$, $\Delta \tau_{j}$ is the epoch delay of each decentralized epoch coordinate $x_{j}$ relative to the current observation epoch coordinate $x_{r}$, and $\vartheta_{j}$ is the variation coefficient of each decentralized epoch coordinate $x_{j}$ relative to the current observation epoch coordinate $x_{r}$, and $j\in T,j=t-\left[\frac{T}{2}\right],\ldots ,0,\ldots ,t+\left[\frac{T}{2}\right]$, the weighting coefficient of the decentralized epoch coordinate $x_{j}$ in the current epoch r can be calculated by the following formula:

(11) $$\{\begin{array}{c} \vartheta_{j}=\frac{{\left(\Delta \tau_{j}-\bar{\tau }\right)}^{2}}{\bar{\tau }}\\ \bar{\tau }=\frac{\sum_{i=t-\left[\frac{T}{2}\right]}^{t+\left[\frac{T}{2}\right]}\Delta \tau_{j}}{T}\\ \alpha_{j}=\frac{\vartheta_{j}}{\sum_{j=t-\left[\frac{T}{2}\right]}^{t+\left[\frac{T}{2}\right]}\vartheta_{j}} \end{array}.$$

In the value domain, the difference between each decentralized epoch coordinate $x_{k}$ and the current observation epoch coordinate $x_{r}$ is calculated, and the weighting coefficient are assigned to the respective decentralized epoch coordinate $x_{k}$ according to the magnitude of the difference, and, the smaller the difference, the greater the influence of similarity effect, the greater the weight of the corresponding decentralized epoch coordinate $x_{k}$. Let $\bar{\mu }$ be the average of the magnitude of the difference of the decentralized epoch coordinate $x_{k}$, $\Delta \mu_{k}$ is the difference of the each decentralized epoch coordinate $x_{k}$ with respect to the current observation epoch coordinate $x_{r}$, and $\vartheta_{k}$ is the variation coefficient of the each decentralized epoch coordinate $x_{k}$. Then, the weighting coefficient of the decentralized epoch coordinate $x_{k}$ in the current epoch r can be calculated by the following formula:

(12) $$\{\begin{array}{c} \vartheta_{k}=\frac{{\left(\Delta \mu_{k}-\bar{\mu }\right)}^{2}}{\bar{\mu }}\\ \bar{\mu }=\frac{\sum_{k=1,}^{N_{r}}\Delta \mu_{k}}{N_{r}}\\ \beta_{k}=\frac{k}{\sum_{k=1,}^{N_{r}}\vartheta_{k}} \end{array}.$$

#### 3.2.4. Calculating the Time Domain Component $X_{r}^{T}$ and the Value Domain Component $X_{r}^{N_{r}}$

Then, we substitute T, $N_{r}$, $x_{j},x_{k},\;\alpha_{j}$*,* $\beta_{k}$ into Equation (5), the current observation epoch coordinate $x_{r}$ is subjected to moving weighted averaging processing in the time domain and in the value domain, respectively, and the time domain component $X_{r}^{T}$ and the value domain component $X_{r}^{N_{r}}$ of the current epoch coordinate are obtained.

#### 3.2.5. Calculating the Smoothed Observation Epoch $\hat{X_{r}}$

Next, the time domain component $X_{r}^{T}$ and the value domain component $X_{r}^{N_{r}}$ of the current observation epoch r are comprehensively weighted:

(13) $$\hat{X_{r}}=\gamma_{T}X_{r}^{T}+\gamma_{N_{r}}X_{r}^{N_{r}},$$

where $\gamma_{T}$ is the weighting coefficient of the time domain component, $\gamma_{N_{r}}$ is the weighting coefficient of the value domain component, and $\hat{X_{r}}$ is the new value smoothed in the current observation epoch r.

In the current epoch *r*, the difference of the time domain component and the current observation epoch coordinate $x_{r}$, and the difference of the value domain component and the current observation epoch coordinate $x_{r}$ are calculated, respectively, and the values of the corresponding components are weighted according to the magnitude of the difference.

Assuming that the average value of the current epoch coordinate $x_{r}$ in two-dimensional space is $\bar{\Upsilon }$, the variation coefficient of the time domain component is $\vartheta_{T}$, and the variation coefficient of the value domain is $\vartheta_{N_{r}}$. Then, the weighting coefficients of the time domain component and the value domain component of the current observation epoch r are calculated by the following formula:

(14) $$\{\begin{array}{c} \vartheta_{T}=\frac{{\left(\Delta X_{T}-\bar{\Upsilon }\right)}^{2}}{\bar{\Upsilon }}\\ \vartheta_{N_{r}}=\frac{{\left(\Delta X_{N_{r}}-\bar{\Upsilon }\right)}^{2}}{\bar{\Upsilon }}\\ \gamma_{T}=\frac{\vartheta_{T}}{\vartheta_{T}+\vartheta_{N_{r}}}\\ \gamma_{N_{r}}=\frac{\vartheta_{N_{r}}}{\vartheta_{T}+\vartheta_{N_{r}}} \end{array},$$

where $\bar{\Upsilon }=\frac{X_{r}^{T}+X_{r}^{N_{r}}}{2}$, $\Delta X_{T}=X_{r}^{T}-\bar{\Upsilon },\Delta X_{N_{r}}=X_{r}^{N_{r}}-\bar{\Upsilon }$. Finally, we substitute the obtained $\gamma_{T}$ and $\gamma_{N_{r}}$ into Equation (13) to obtain the new value $\hat{X_{r}}$ of the current observation epoch r, which is the smoothed observation epoch coordinate.

Following the steps as above, this method is expected to solve the edge effect and achieve the purpose of edge preservation and denoising, but using this method will retain too much high frequency information, so that the white noise in high frequency will not be completely eliminated, so this paper will add wavelet packet transform based on this method to further eliminate the white noise in high frequency.

## 4. Time-Frequency Conversion by WPT

### 4.1. The Theory of WPT

From the introduction, we have known that wavelet packet transform provides a more sophisticated analysis of the signal. Wavelet packet transform divides the time-frequency space into more detail, and it has higher resolution for the high-frequency part of the signal than the binary wavelet transform. Moreover, based on the theory of wavelet analysis, it introduces the concept of optimal basis selection. That is, after the frequency band is divided into multiple levels, according to the characteristics of the analyzed signal, the optimal basis function is adaptively selected to match the signal to improve the signal analysis capability.

In order to further eliminate the white noise of the high frequency part of the smoothed DD coordinate residual sequences, which wavelet packet transform is applied to decompose, the wavelet packet decomposition algorithm [21] can be indicated as Equation (15):

(15) $$\{\begin{array}{c} d_{M}^{2\omega -1}=\sum_{k\in Z}H\left(k-2i\right)d_{M-1}^{\omega }\\ d_{M}^{2\omega }=\sum_{k\in Z}G\left(k-2i\right)d_{M-1}^{\omega } \end{array},$$

where $d_{M}^{2\omega -1}$ denotes the 2ω − 1 wavelet packet coefficient of the *M*-th layer, $d_{M-1}^{\omega }$ represents the *ω*-th wavelet packet coefficient on the M-1 layer, and $p_{M}^{2\omega }$ represents the 2*ω*-th wavelet packet coefficients on the *M*-th layer, G is the decomposition filter relation to scale function, H is the decomposition filter of wavelet function, and $M\geq 1,\;i=1\sim I,\omega =1\sim 2^{M}$.

The wavelet packet reconstruction algorithm [21] can be indicated as Equation (16):

(16) $$d_{M}^{\omega }=2\left[\sum_{k\in Z}h\left(i-2k\right)d_{M+1}^{2\omega -1}+\sum_{k\in Z}g\left(i-2k\right)d_{M+1}^{2\omega }\right],$$

where $d_{M+1}^{2\omega -1}$ represents the 2ω−1 wavelet packet coefficient on the M+1 layer, $d_{M+1}^{2\omega }$ indicates the 2ω wavelet packet coefficients on the J+1 layer, g is the reconstruction filter related to scaling function, and h is the reconstruction filter related to wavelet function.

From the perspective of time-frequency conversion, the DD coordinate residual sequences after the first WPT decomposition could be converted into two equal frequency bands by Equation (15), i.e., $\left(0\sim \frac{N_{f}}{2}\right)$ and $\left(\frac{N_{f}}{2}\sim N_{f}\right)$, where $N_{f}$ is Nyquist frequency. The WPT coefficients $d_{M-1}^{\omega }$ are divided into $d_{M}^{2\omega -1}$ and $d_{M}^{2\omega }$ as well. Moreover, both $\left(0\sim \frac{N_{f}}{2}\right)$ and $\left(\frac{N_{f}}{2}\sim N_{f}\right)$ can be divided continuously.

**Figure 1:**
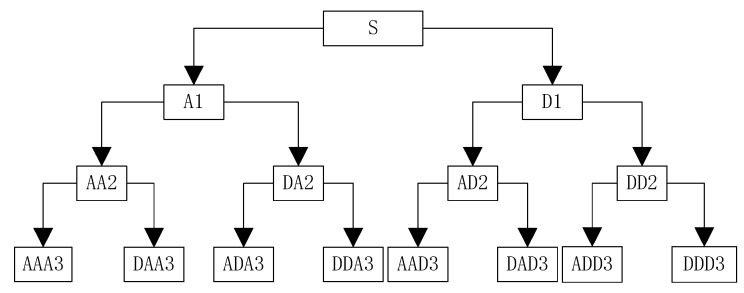
Wavelet packet decomposition tree.

An example of three level decomposition of WPT is shown as Figure 1.

In Figure 1, A represents a low frequency, D represents a high frequency, and the serial number at the end represents the number of layers of wavelet decomposition.

The decomposition relationship can be expressed as Equation (17):

(17) S=AAA3+DAA3+ADA3+DDA3+AADD3+DAD3+ADD3+DDD3.

After the DD coordinate residual sequences are decomposed according to the structure shown in Figure 1, using the thresholding denoising mentioned in the Section 4.4, the wavelet packet coefficients that satisfy the condition will be used to reconstruct the compressed DD coordinate residual sequences while others will be set to zero.

### 4.2. The Theory of Correlation Analysis

In view of the strong correlation of multipath in the same time between the adjacent days in static positioning, though the smoothed DD coordinate residual sequences still contain residual white noise, but the multipath error is dominant, which contributes to strong correlation between the smoothed DD coordinate residual sequences.

We have the decomposed DD coordinate residual sequences in Section 3.1, which can be applied to correlation analysis for determining the best decomposition level of the WPT.

In the three consecutive days, regard one day as the reference day and the rest as the adjacent days.

A is the wavelet packet coefficient of the low-frequency part of the decomposed DD coordinate sequences of the reference day, and B is the wavelet packet coefficient of the corresponding low-frequency part of the decomposed DD coordinate sequence of the adjacent day, and $\rho_{AB}$ is the cross-correlation coefficient.

The formula for calculating the correlation coefficient can be expressed as Equation (18):

(18) $$\rho_{\mathrm{AB}}=\frac{cov\left(A,B\right)}{\sqrt{D\left(A\right)}\sqrt{D\left(B\right)}},$$

where $cov\left(A,B\right)=\sum_{\omega =1}^{2M}\left(a_{\omega }-u_{a}\right)\left(b_{\omega }-u_{b}\right)$ is the covariance of variables A and B, $D\left(A\right)=\sum_{\omega =1}^{2M}{\left(a_{\omega }-u_{a}\right)}^{2}$ is the variance of the variable A, $D\left(B\right)=\sum_{\omega =1}^{2M}{\left(b_{\omega }-u_{b}\right)}^{2}$ is the variance of the variable B, $u_{a}=\frac{1}{2M}\sum_{\omega =1}^{2M}a_{\omega }$ is the average value of the variable A, $u_{b}=\frac{1}{2M}\sum_{\omega =1}^{2M}b_{\omega }$ is the average value of the variable B.

The magnitude of $\rho_{\mathrm{AB}}$ reflects the correlation degree between the analyzed sequences. The closer the correlation coefficient $\left|\rho_{\mathrm{AB}}\right|$ is to 1, the closer A and B are to linear correlation, and the greater the correlation of A and B.

However, due to the influence of residual white noise, satellite visible conditions and the distance between the antenna of the monitoring station and the reflecting surface, the correlation coefficient of the smoothed DD coordinate residual sequences between adjacent days becomes smaller.

### 4.3. The Method for Determining the Best Decomposition Level of the WPT

Determining the best decomposition level of the WPT that is related to the validity of multipath extraction, if there are too many decomposition layers, the useful information in the smoothed DD coordinate residual sequences will be lost. If the number of decomposition layers is too small, the white noise in the smoothed DD coordinate residual sequences cannot be eliminated absolutely.

The method based on the correlation analysis for determining the best decomposition level of the WPT can be described as follows:

Step1: Calculating the cross-correlation coefficient of the smoothed DD coordinate residual sequences between the reference day and other adjacent days, for any reference day, and selecting the smoothed DD coordinate residual sequences that have the largest cross correlation coefficients.

Step2: Performing M-layers decomposition on the set of the smoothed DD coordinate residual sequences obtained in step 1 by using the WPT algorithm, and calculating the cross-correlation coefficient of the wavelet packet coefficients of the approximate part (low-frequency part) of the each decomposition layer between the smoothed DD coordinate residual sequences obtained in step 1.

Step3: Determining whether the target layer J is smaller than M, where M is the preset number of decomposition layers, and the target layer J is the number of layers in which the maximum value of the cross-correlation coefficient is located.

If yes, determining the target layer J be the best decomposition level of the WPT;

If not, the value of M is incremented by one, and the process returns to step 3 until the target layer J is smaller than M. By this way, we can figure out the best decomposition level of the WPT that can be applied to analyze the smoothed DD coordinate residual sequences in the optimal state.

### 4.4. The Principle of Wavelet Packet Threshold Denoising

We have the decomposed DD coordinate residual sequences with the best decomposition level of the WPT and the wavelet packet coefficients obtained should be denoised by thresholding for reconstructing the DD coordinate residual sequences.

The process of denoising by thresholding is carried out by comparing the magnitude of the wavelet packet coefficients $d_{i}$ with a threshold λ. The process can be described in two aspect: one is the choice of the threshold function F and the other is the choice of the threshold parameter λ.

There are three choices of threshold function F including hard thresholding $F_{\lambda }^{H}\left(d_{i}\right)$, soft thresholding $F_{\lambda }^{S}\left(d_{i}\right)$ and quantitative thresholding $F_{\lambda }^{Q}\left(d_{i}\right)$ [28]:

(19) $$F_{\lambda }^{H}\left(d_{i}\right)=\{\begin{array}{ll} 0, & \left|d_{i}\right|<\lambda \\ d_{i}, & \left|d_{i}\right|\geq \lambda \end{array},$$

(20) $$F_{\lambda }^{S}\left(d_{i}\right)=\mathrm{sign}\left(d_{i}\right)\left(\left|d_{i}\right|-\lambda \right),\;\;\left|d_{i}\right|<\lambda ,$$

(21) $$F_{\lambda }^{Q}\left(d_{i}\right)=\{\begin{array}{ll} 0, & \left|d_{i}\right|<Ρ\\ d_{i}, & \left|d_{i}\right|\geq Ρ \end{array},$$

where $d_{i}$ denotes the wavelet coefficients, and Ρ is the value for which a certain percentage of coefficients $d_{i}$ is eliminated [17].

In this paper, the hard thresholding function is chosen for denoising the wavelet packet coefficients, which had been proved be the most suitable function for GPS applications [17].

As for the choice of the threshold parameter λ, Donoho (1995) had proposed the universal threshold [29]:

(22) $$\lambda =\sqrt{2\sigma^{2}\log\left(n\right)},$$

where n denotes the number of samples in white noise vector N and σ is the white noise level and needs to be estimated in each DD coordinate residual sequence. For determining the value of σ, Donoho and Johnstone (1994) proposed the following estimator [30]:

(23) $$\sigma =\frac{\mathrm{Median}\left[\left|N\right|\right]}{0.6745}.$$

The noise vector N in the first level of decomposition layers is used for determining the value of σ.

Applying the thresholding method mentioned as above, all of the wavelet packet coefficients are denoised, which contribute to the reconstruction of the DD coordinate residual sequences and obtain the noiseless multipath.

## 5. The Overall Program

**Figure 2:**
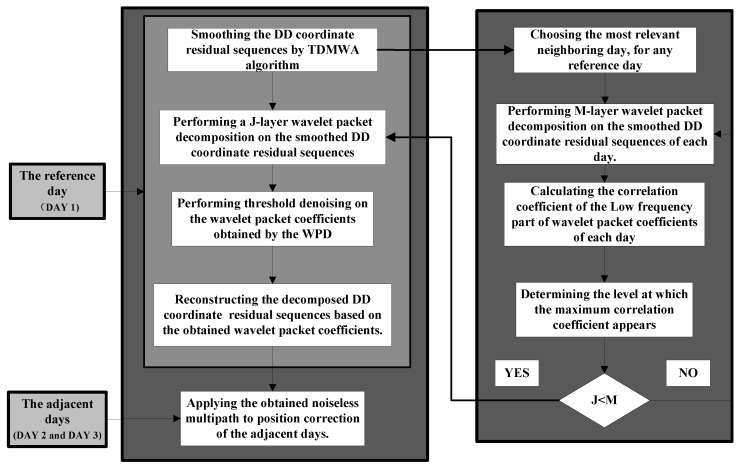
The overall program of mitigating the multipath in GPS static high-precision positioning.

The overall program of mitigating the multipath in GPS static high-precision positioning is shown in Figure 2.

## 6. Verify the Feasibility of the Algorithm under Simulated Conditions

The DD coordinate residual sequences is simulated with the following model:

(24) $$S\left(t\right)=\cos\left(\frac{2\pi t}{1200}\right)+\sin\left(\frac{2\pi t}{900}\right)+\sin\left(\frac{2\pi t}{300}\right)+e\left(t\right),$$

where S(t) consists of a cosine signal with a period of 1200 s and two sinusoidal signals with periods of 900 s and 300 s which represents typical GPS multipath wavelengths, and e(t) denotes a Gaussian white noise sequence.

Take the noise level of e(t) as N (0, 1.5^2^). The data sampling rate is 1 s, and the sample size is 6000.

The simulated signal is denoised by WT transform, the TDMWA algorithm and WP-TD algorithm, respectively. DAUB8 is taken as the wavelet function, and we assume that the best decomposition level is 5.

**Figure 3:**
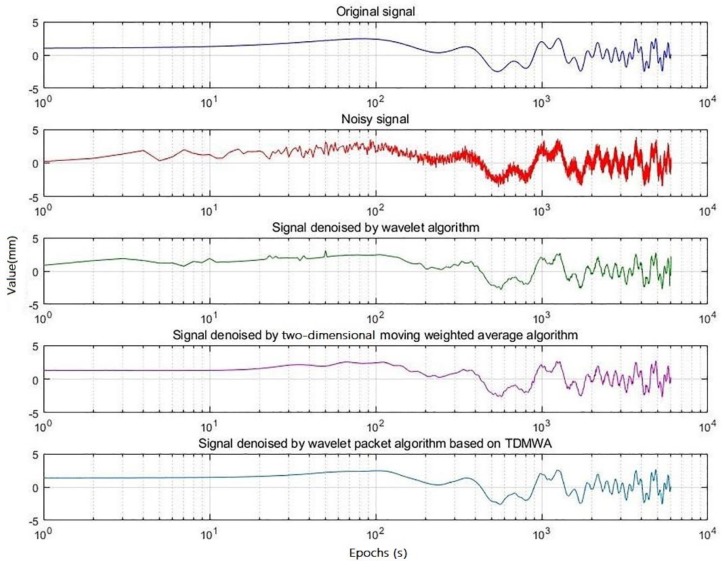
The signals denoised by the three different algorithms.

**Figure 4:**
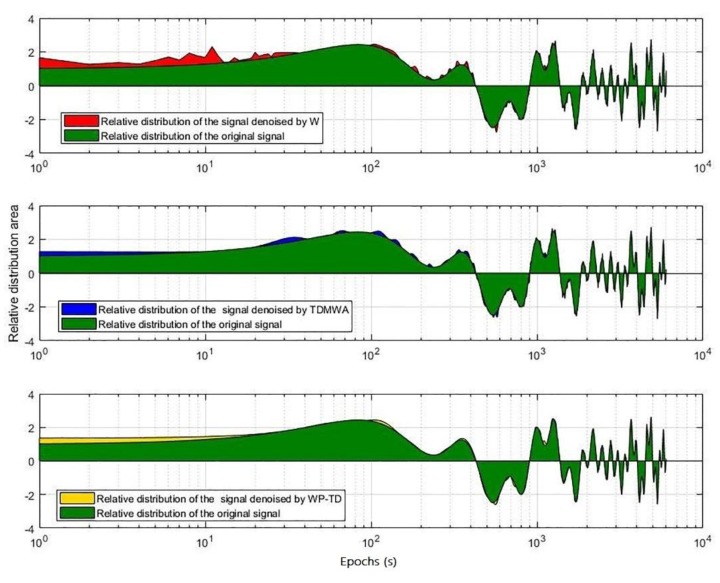
The relative distribution of the signal denoised by the three different algorithms with the original signal.

The denoising result is shown in Figure 3, and the relative distribution of the signal denoised by the three different algorithms with the original signal is shown in Figure 4.

**Figure 5:**
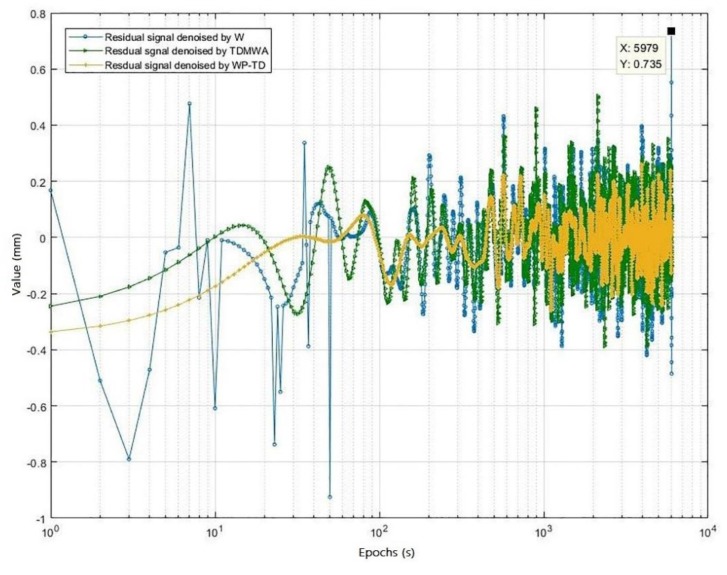
The residual signals generated by the three different algorithms.

The residual signal generated by the three different algorithms is shown in Figure 5.

From Figure 4 and Figure 5, we can visually see that the signal denoised by the WP-TD algorithm better restores the original signal. The denoised result of the signal processed using the TDMWA algorithm is also slightly better than the traditional WT algorithm. In addition, the signal denoised using the WT algorithm has an edge effect, but the signal denoised using the TDMWA algorithm does not appear.

The signals denoised by the above three algorithms and the residual signals generated are analyzed quantitatively. The analysis indicators include the root mean square (RMS) value of the noise part of denoised signal $N_{RMS}$, the RMS value of the signal part of the denoised signal $S_{RMS}$ and the correlation coefficient ρ between the denoised signal and the original signal. In order to scientifically evaluate the performance of the three algorithms, simulation analysis was carried out in four different Gaussian white noise simulation environments. The results are shown in Table 1, Table 2 and Table 3.

On one hand, it can be seen from Table 1, Table 2 and Table 3 that the correlation coefficients of the three algorithms mostly exceed 0.95 at different noise levels, indicating that all of them can restore the information of the original very well. However, the value of $S_{RMS}$ of the WP-TD algorithm is relatively optimal at different noise levels, and its correlation coefficient is greater than 0.98.

On the other hand, the value of $N_{RMS}$ of the three algorithms are basically equivalent while there is some difference in the value of $S_{RMS}$. According to the tables, as the noise level increases, the value of $S_{RMS}$ of the WT algorithm and the TDMWA algorithm increase significantly compared with WP-TD algorithm. However, at high noise levels, the TDMWA algorithm increases slowly while the WT algorithm is relatively fast, and its $S_{RMS}$ is still superior to the WT algorithm.

In summary, we can draw such a conclusion that the WP-TD algorithm can denoise the noise signal more effectively, and can effectively weaken the influence of the edge effect in signal filtering.

## 7. Analyze the Performance of the Algorithm in a Measured Environment

In order to further verify the ability of the WP-TD algorithm to eliminate noise in the actual environment, we established a GPS data acquisition system in the laboratory and conducted experiments in a multipath environment.

### 7.1. Set up GPS Data Acquisition System

**Figure 6:**
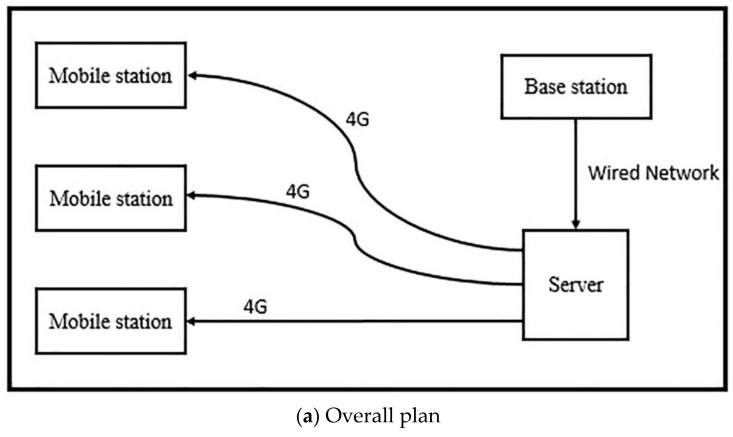

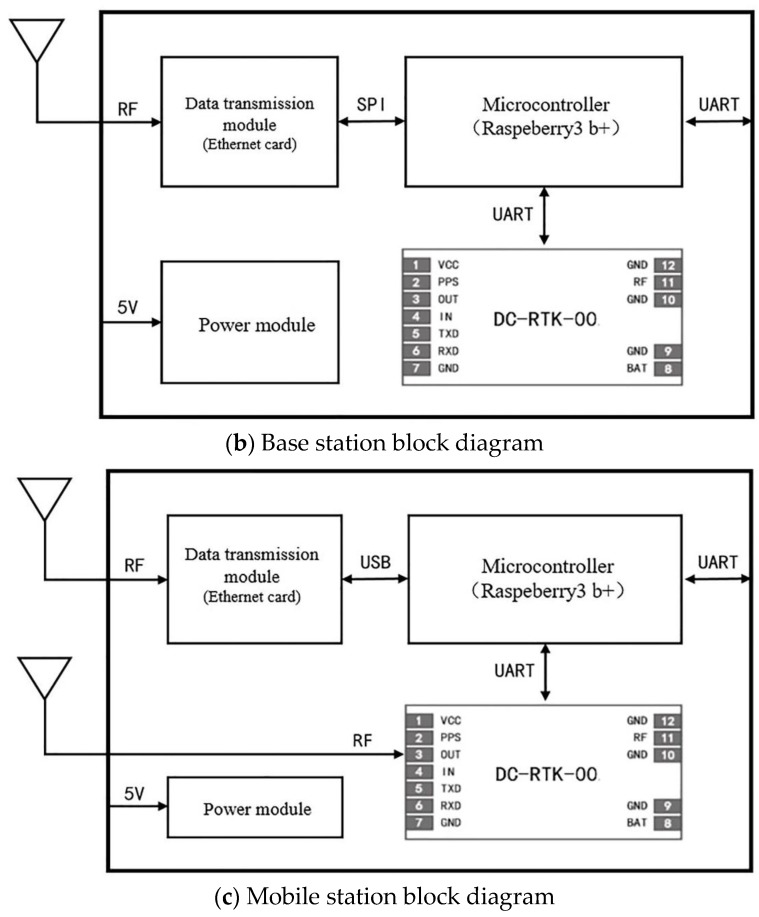
Data acquisition system.

**Figure 7:**
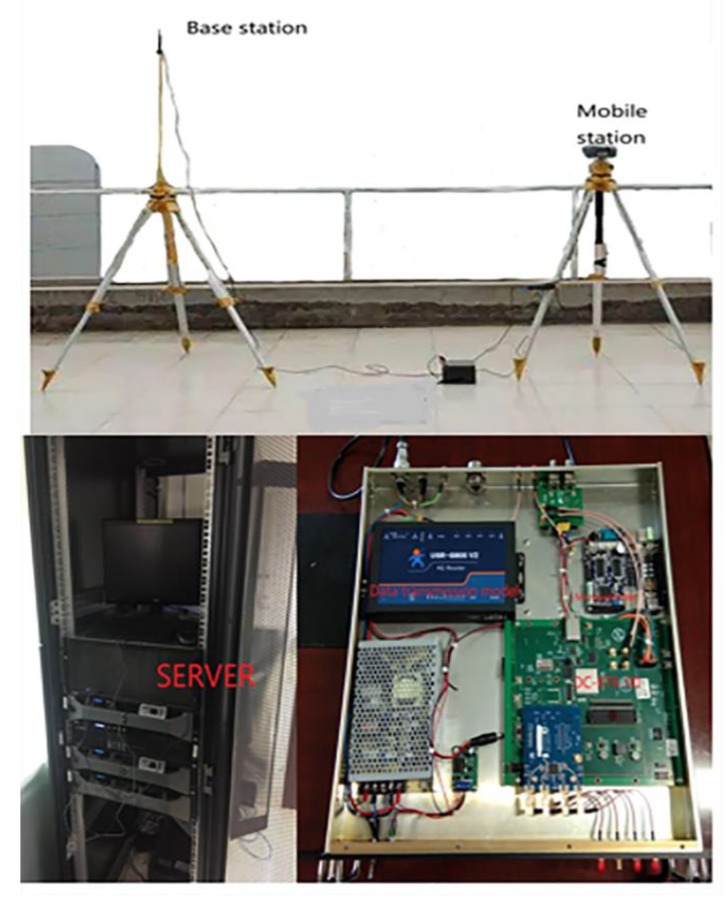
Scene map and physical device diagram.

The block diagram of the overall scheme and key modules of the positioning data acquisition system are shown in Figure 6 and Figure 7, which shows the scene map and physical device diagram.

In this system, the base station and mobile station are all equipped with a DC-RTK-00 Beidou/GPS/Galileo three-mode single-frequency RTK module, a power model (5 V), a microcontroller (Raspberry3 b+) and a data transmission module (Ethernet card/4G card). The base station sends the differential data to the server over the wired network and is cached by the lab server. The mobile station accesses the server through the 4G network to obtain differential data, and, at the same time, the mobile station equipped with the 4G network card transmits the positioning result back to the lab server.

### 7.2. Data Collection and Processing

The fixed point is located at the top of the research building of Beijing University of Posts and Telecommunications. The settings of related parameters of data acquisition are shown in Table 4.

The DD coordinate residual sequences to be processed are part of the elevation data sequence measured of the fixed point for three consecutive days in a multipath environment.

**Figure 8:**
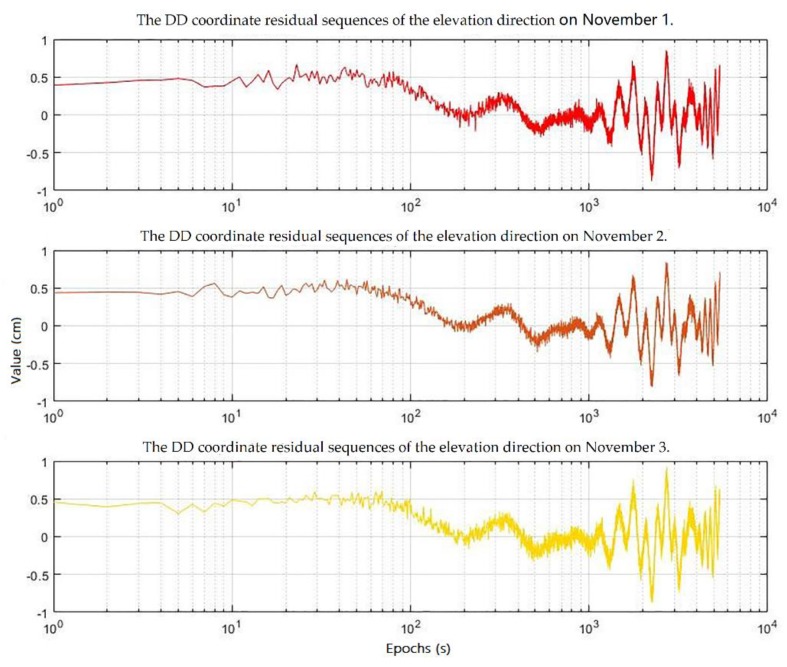
Original DD coordinate residual sequences of the elevation direction for the three consecutive days.

Figure 8 shows the DD coordinate residual sequences of the elevation direction for three consecutive days. As can be seen from Figure 8, under the influence of the observation noise, the DD coordinate residual sequences for three consecutive days still have significant repeatability.

According to the overall program of mitigating the multipath in GPS static high-precision positioning established in this paper, we need to smooth the DD coordinate residual sequences first using the TDMWA algorithm.

Secondly, we need to analyze the correlation of the DD coordinate sequences residuals for three consecutive days and determine the best decomposition level of WPT based on the method proposed in this paper.

**Figure 9:**
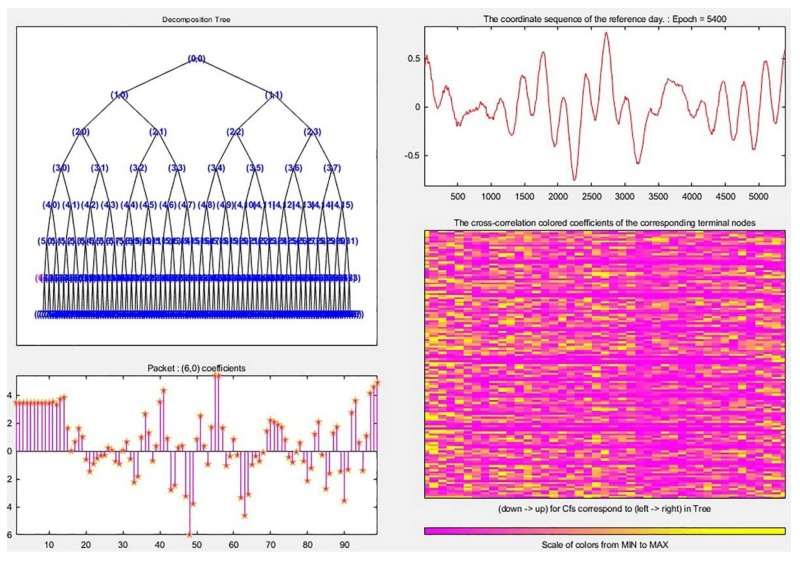
The process of determining the best decomposition level of WPT.

The process of determining the best decomposition level of WPT is shown in Figure 9.

From right to left, and from top to bottom are the DD coordinate residual sequence of the reference day preprocessed by the TDMWA algorithm, the seven-layer decomposition tree structure of the DD coordinate residual sequence of the reference day, the cross-correlation colored coefficients of the corresponding nodes and the value of the first wavelet packet coefficient of the decomposed DD coordinate residual sequence of the reference day located on the sixth layer. It can be seen from the distribution of the colored coefficients in the figure that the larger values of the correlation coefficient are mostly distributed in the sixth layer and the fifth layer, and the maximum value appears in the sixth layer.

According to the judgment conditions in the method proposed in this paper, we can determine that the best denoise result can be obtained when the decomposition level of the wavelet packet is six layers.

Next, we perform a six-layer wavelet packet decomposition on the smoothed DD coordinate residual sequences and reconstruct the decomposed DD coordinate residual sequences based on the wavelet packet coefficients obtained by threshold denoising.

Finally, we can obtain almost noiseless multipath, which can be applied to correct the position sequences of the adjacent days.

**Figure 10:**
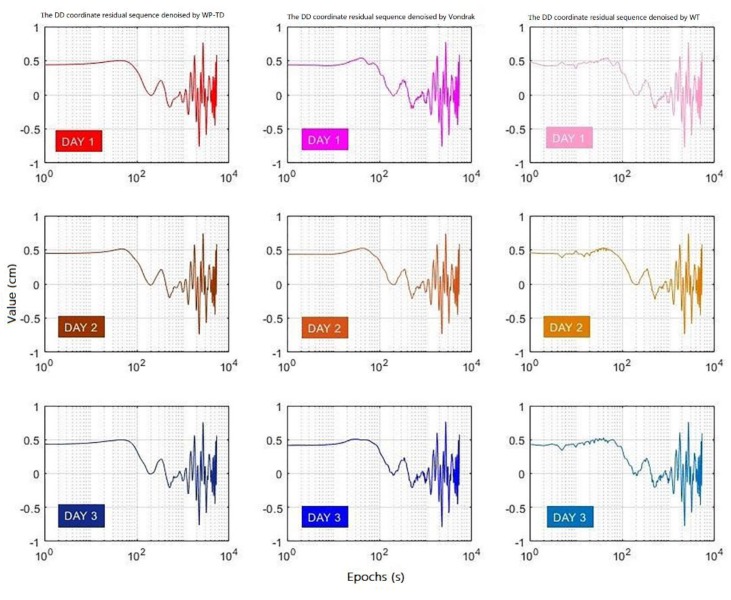
The DD coordinate residual sequences denoised by the three algorithms for three consecutive days.

**Figure 11:**
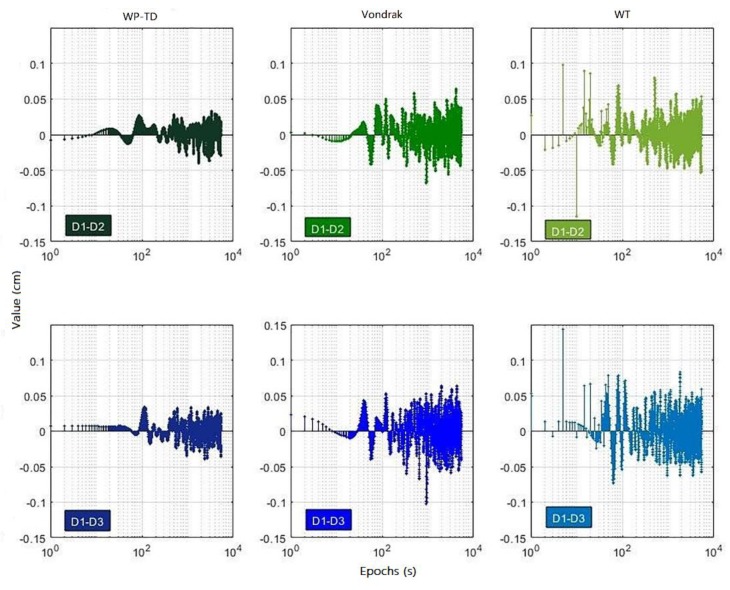
The difference of DD coordinate residual sequences denoised by the three algorithms.

**Figure 12:**
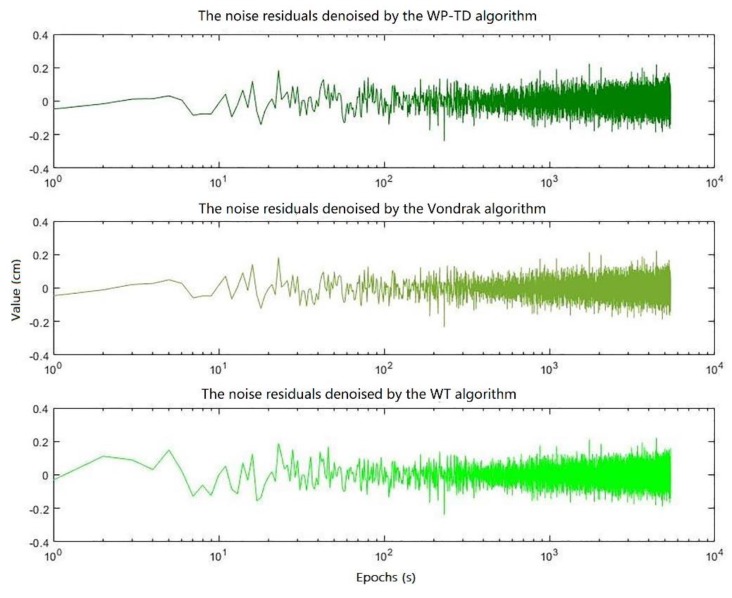
Noise residuals denoised by the three algorithms on the first day.

The DD coordinate residual sequences denoised by the WP-TD algorithm, Vondrak algorithm and WT algorithm for three consecutive days are shown in Figure 10, and Figure 11 shows the difference of denoised DD coordinate residual sequences for the second day and the third day compared to the first day, respectively, and Figure 12 shows the noise residuals denoised by the three algorithms on the first day.

It can be seen from the Figure 10 and Figure 11 that the difference of DD coordinate residual sequence processed by the WP-TD algorithm is much smaller than the WT algorithm and the Vondrak algorithm, which shows that applying the DD coordinate residual sequence denoised by the WP-TD algorithm to correct the position sequence of the adjacent day will be more precise.

Moreover, the DD coordinate residual sequence has a larger extremum at the end of sequence, which was denoised by the WT algorithm without the preprocessing of TDMWA algorithm, while the DD coordinate residual sequence presents a smooth curve at the end of the sequence which is preprocessed by the TDMWA algorithm before being denoised by the WPT algorithm.

### 7.3. Data Analysis

In order to further compare the performance for extracting multipath of the three algorithms in the actual environment, the RMS value of the DD coordinate residual sequences before and after filtering, the RMS value of the DD coordinate residual sequences of the adjacent days corrected by the almost noiseless multipath obtained in the reference day and the correlation coefficient of the DD coordinate residual sequence of the three days are used as the evaluation indicator.

The comparison results of the three algorithms are shown in Table 5, Table 6 and Table 7, respectively.

First of all, it can be seen from the results in Table 5 that the RMS value of the DD coordinate residual sequence after filtering by the WP-TD algorithm is basically equivalent to the RMS value of the DD coordinate residual sequence after filtering by the Vondrak algorithm, but both are slightly better than the RMS value of the DD coordinate residual sequence after filtering by the WT algorithm. In addition, the noise residuals denoised by the three algorithms on the first day are not much different as shown in Figure 12, indicating that the observation noise (white noise) is only a small part of the DD coordinate residuals sequence, and multipath dominates.

Secondly, the results in Table 6 show that the GPS multipath of the fixed point is highly repetitive, and the correlation coefficient increases after filtering observation noise. Moreover, the performance of filtering of the WP-TD algorithm is better than WT algorithms and Vondrak algorithms more or less. Through calculation, we can know that the correlation of the DD coordinate residual sequence of the three days filtered by WP-TD is 3.02% and 1.78% higher than that of WT algorithms and Vondrak algorithms, respectively.

Last but not least, according to the data in Table 7, after correcting by the denoised multipath of the reference day (Day 1) which was extracted by the WT algorithms, the Vondrak algorithms, and the WP-TD algorithm, we can figure out that the accuracy of the position sequence of the adjacent days (Day 2 and Day 3) is increased by 57.47%, 65.98%, and 71.61%, respectively, compared with before correction, which further verified that the WP-TD algorithm can obtain an more accurate multipath correction model.

## 8. Conclusions

The WP-TD algorithm is proposed, in which the method of determining the optimal decomposition level of WPT is given, which can avoid the redundancy and loss of information after signal reconstruction to the greatest extent.

The WP-TD algorithm is proposed, which makes use of the TDMWA algorithm, and is proven to have good performance that weakens the influence of the edge effect through simulation analysis and measured data analysis.

The WP-TD algorithm is proposed, which uses the WPT to divide the signal frequency band into multiple layers, further decomposes the high frequency part without subdivision in the traditional WT algorithm, and eliminates the observation noise in the high frequency part.

The WP-TD algorithm is proposed, which applies the denoised multipath of the reference day to correct the position sequence of the adjacent days, improving positioning accuracy by 14.4%, compared to the traditional WT algorithm.

In summary, the WP-TD algorithm is proposed, which can perform well for eliminating the multipath in GPS static high-precision positioning.

## Figures and Tables

**Table 1:** Performance indicators of the WT algorithm in different Gaussian white noise simulation environments.

Noise Level	0.5	1.5	2.5	3.5
$S_{RMS}$	0.1382	0.2648	0.3232	0.3631
$N_{RMS}$	0.4912	1.4514	2.4623	2.9827
ρ	0.9886	0.9639	0.9624	0.9593

**Table 2:** Performance indicators of the TDMWA algorithm in different Gaussian white noise simulation environments.

Noise Level	0.5	1.5	2.5	3.5
$S_{RMS}$	0.1268	0.2231	0.2638	0.2985
$N_{RMS}$	0.4889	1.4485	2.46884	2.9816
ρ	0.9892	0.9802	0.9742	0.9698

**Table 3:** Indicators of the WP-TD algorithm in different Gaussian white noise simulation environments.

Noise Level	0.5	1.5	2.5	3.5
$S_{RMS}$	0.0887	0.1401	0.2022	0.2436
$N_{RMS}$	0.4722	1.4353	2.46841	2.9707
ρ	0.9998	0.9882	0.9832	0.9801

**Table 4:** The setting of data acquisition parameters.

Main Parameters	Value
Receive signal type	GPS L1
Sampling frequency (HZ)	1
Baud rate (bps)	115,200
Elevation mask angle (deg)	20.000
SNR mask (dB)	30.000
Baseline length (km)	<3
Sampling time	9:00 p.m.–10:30 p.m. (1–3 November)
Total epoch	5400

**Table 5:** RMS value of the DD coordinate residual sequences before and after filtering by the three algorithms (mm).

Time	Before Filtering	WT	Vondrak	WP-TD
D1	16.4422	16.3224	16.0088	15.8628
D2	17.3895	17.3347	16.7655	16.6834
D3	16.9872	16.7825	16.4456	16.2218

**Table 6:** Correlation coefficient of the DD coordinate residual sequence of the three days after being denoised by the three algorithms.

Time	Before Filtering	WT	Vondrak	WP-TD
D1-D2	0.9388	0.9586	0.9722	0.9873
D2-D3	0.9236	0.9521	0.9636	0.9822
D3-D1	0.9248	0.9549	0.9647	0.9826

**Table 7:** The RMS value of the DD coordinate residual sequences of the adjacent days corrected by the almost noiseless multipath obtained in the reference day (mm).

Time	Before Filtering	WT	Vondrak	WP-TD
D2	15.3476	6.5452	5.4543	4.5343
D3	16.8248	7.1347	5.4668	4.5839
